# Plasma suPAR levels are associated with mortality, admission time, and Charlson Comorbidity Index in the acutely admitted medical patient: a prospective observational study

**DOI:** 10.1186/cc11434

**Published:** 2012-07-23

**Authors:** Thomas Huneck Haupt, Janne Petersen, Gertrude Ellekilde, Henrik Hedegaard Klausen, Christian Wandall Thorball, Jesper Eugen-Olsen, Ove Andersen

**Affiliations:** 1 Clinical Research Centre, Copenhagen University Hospital Hvidovre, Kettegaard Alle 30, Hvidovre, 2650, Denmark; 2 The Acute Medical Unit, Copenhagen University Hospital Hvidovre, Kettegaard Alle 30, Hvidovre, 2650, Denmark

## Abstract

**Introduction:**

Soluble urokinase plasminogen activator receptor (suPAR) is the soluble form of the membrane-bound receptor (uPAR) expressed predominantly on various immune cells. Elevated plasma suPAR concentration is associated with increased mortality in various patient groups, and it is speculated that suPAR is a low-grade inflammation marker reflecting on disease severity. The aim of this prospective observational study was to determine if the plasma concentration of suPAR is associated with admission time, re-admission, disease severity/Charlson Comorbidity Index Score, and mortality.

**Methods:**

We included 543 patients with various diseases from a Danish Acute Medical Unit during a two month period. A triage unit ensured that only medical patients were admitted to the Acute Medical Unit. SuPAR was measured on plasma samples drawn upon admission. Patients were followed-up for three months after inclusion by their unique civil registry number and using Danish registries to determine admission times, readmissions, International Classification of Diseases, 10^th ^Edition (ICD-10) diagnoses, and mortality. Statistical analysis was used to determine suPAR's association with these endpoints.

**Results:**

Increased suPAR was significantly associated with 90-day mortality (4.87 ng/ml in survivors versus 7.29 ng/ml in non-survivors, *P *< 0.0001), higher Charlson Score (*P *< 0.0001), and longer admission time (*P *< 0.0001), but not with readmissions. The association with mortality remained when adjusting for age, sex, C-reactive protein (CRP), and Charlson Score. Furthermore, among the various Charlson Score disease groups, suPAR was significantly higher in those with diabetes, cancer, cardiovascular disease, and liver disease compared to those without comorbidities.

**Conclusions:**

SuPAR is a marker of disease severity, admission time, and risk of mortality in a heterogeneous cohort of patients with a variety of diseases. The independent value of suPAR suggests it could be of value in prognostic algorithms.

## Introduction

At acute medical units, the crucial decision is whether to admit or discharge a patient. Currently, this decision is based on a clinical examination of the patient and on various diagnostic tests. A reliable prognostic algorithm can aid in deciding whether to run a diagnostic panel, as well as whether to discharge or admit a patient. A new biomarker that may be useful in prognostic algorithms is soluble urokinase plasminogen activator receptor (suPAR). SuPAR is the soluble form of urokinase plasminogen activator receptor (uPAR), a receptor mainly expressed on various immunologically active cells such as macrophages, neutrophils, and activated T lymphocytes, as well as on endothelial cells [1]. uPAR is involved in plasminogen activation, cell adhesion and migration, all central aspects of inflammatory processes, and it is upregulated in response to immune activation [2]. Plasma levels of suPAR have been shown to be a risk marker in patients with various infectious diseases [3-5] and in the intensive care settings [6-8]. High levels of suPAR are associated with increased risk of early mortality [9] and the suPAR level is thought to reflect disease severity [6].

The Charlson Comorbidity Index Score (Charlson Score) is calculated on the basis of patient diagnoses coded according to the International Classification of Diseases, 10^th ^Edition (ICD-10), which are listed upon discharge from the hospital. A weighted score is assigned to each of 17 comorbidity groups based on the relative risk of 10-year mortality. The cumulative score of these 17 groups has been validated as a prognostic indicator in large patient groups, with higher scores indicating a higher risk of dying. In a recent study by Christensen *et al*., the Charlson Score, along with other administrative parameters, predicted one-year mortality with similar precision as the Simplified Acute Physiology Score (SAPS II) and Acute Physciology and Chronic Health Evaluation (APACHE) [10]. To our knowledge, a possible association between suPAR and the Charlson Score has not been investigated previously.

The association between increased plasma suPAR and increased mortality is documented in patients with HIV or sepsis, and in people from the general population [4,8,11,12], but not in a more diverse patient population. The object of the present study was to evaluate suPAR as a risk marker in a diverse population of medical patients admitted at an acute medical unit, and to determine whether suPAR correlates with the Charlson Score, which would be expected if suPAR reflects disease severity and prognosis in general.

## Materials and methods

### Setting

The Danish health care system provides treatment paid for by taxes for primary care in hospital as well as homecare services; these services are free of charge to all Danish citizens. Furthermore, the hospitals are obliged to provide information about all admissions to the National Patient Registry. The information includes the primary cause of admission and all comorbid diseases, registered using ICD-10. All citizens of Denmark have a unique civil registry number that makes it possible to follow them in various national registers.

### Study population

Patients admitted to the Acute Medical Unit at Hvidovre University Hospital between 4 August and 4 October 2010 were eligible for entry. Patients were included randomly. A triage unit ensured that only medical patients were admitted to the Acute Medical Unit. Exclusion criteria were children under 18 years and patients with no medical condition admitted solely because of acute drug or alcohol intoxication. All included patients gave written informed consent, and the study was approved by the Science Ethics Committee of Copenhagen (protocol no. H-2-2010-031). Patients were followed-up for three months after inclusion using their unique civil registry number. Information about the patients' admissions and ICD-10 diagnoses were extracted from the National Patient Registry. Patients' baseline diagnoses are the main ICD-10 diagnosis from the admission in which they were recruited. This diagnosis reflects what a patient was admitted for, but it is not necessarily the most important diagnosis. Baseline diagnoses were grouped according to ICD-10 chapters into 6 groups with a 7^th ^group ('Other') composed of the 12 ICD-10 chapters with the fewest patients (4 % or less of the total population each). Data on mortality were extracted from the Danish Civil Registry.

### Sample handling

A peripheral venous blood sample was drawn within 24 hours of admission in a standard 4 mL K3 EDTA tube (Cen-Med, East Brunswick, NJ, USA) and centrifuged at 3,000 × *g *for 10 minutes. After centrifugation, EDTA plasma was transferred to a cryotube and stored at −20°C until the plasma suPAR concentration was measured. Blood samples stood at room temperature for up to 16 hours before centrifugation and freezing, but this has previously been shown not to affect the plasma suPAR concentration [13].

### SuPAR measurement

SuPAR was measured using a commercially available sandwich ELISA-kit (ViroGates A/S, Birkerød, Denmark) according to the manufacturer's instructions. Briefly, plasma samples and standards with known suPAR concentrations were added to anti-suPAR-coated microtiter plates and incubated in dilution buffer containing a horseradish peroxidase-conjugated secondary antibody for one hour. After washing to remove any unbound secondary antibody, 3,3',5,5'-tetramethylbenzidine substrate was added, and a color reaction developed for 20 minutes. The color reaction was terminated by the addition of a sulfuric acid stop-solution. The plate was read at 450 nm with wavelength correction at 650 nm. The optical densities of the standards with known concentrations were used to calculate the concentrations of the plasma samples by creating a standard curve. The ELISA-kit manufacturer provided the quantification software. All samples were measured in duplicate, and the mean suPAR concentration of the two measurements was used for analysis. The variance between the two measurements was generally low (mean 2.6%, range 0.0 to 12.4%), and only three samples had a variance >10%. None of these samples were excluded, because all three had a low concentration of suPAR (<7.0 ng/mL), and thus low variance in absolute values.

### Other biomarkers

Plasma concentrations of CRP, creatinine, alanine aminotransferase (ALAT), and albumin and blood concentrations of hemoglobin and leucocytes are routine blood analyses carried out upon admission to Hvidovre University Hospital. Creatinine, CRP, albumin, and ALAT concentrations were measured in blood samples drawn in 4 mL tubes with gel and lithium-heparin using a COBAS 6000 analyzer (Roche Diagnostics, Mannheim, Germany). Blood leucocyte counts and hemoglobin concentrations were measured in blood samples drawn in 4 mL K3EDTA tubes using a Sysmex analyzer (Sysmex XE-5000, Kobe, Japan). Two to five results were missing for each biomarker due to hemolysis or lost samples.

### Charlson Comorbidity Index

We used a SAS macro to calculate the Charlson Score based on the patients' ICD-10 diagnoses from the Danish Patient Registry (for details, see [14]). Only one patient had >4 points, and he was manually recoded to the score 4+ along with the patients who scored 4. For in-depth analysis of suPAR's association with the Charlson Score, the original 17 comorbidity groups were collapsed into seven. Groups 1 to 4 (originally named 'myocardial infarction,' 'congestive heart failure,' 'peripheral vascular disease,' and 'cerebrovascular disease') were collapsed into 'cardiovascular disease.' Similarly, groups 9 and 15 ('mild liver disease' and 'moderate/severe liver disease') were collapsed into 'liver disease.' Groups 10 and 11 ('diabetes without complications' and 'diabetes with complications') were collapsed into 'diabetes.' Groups 14 and 16 ('cancer' and 'metastatic carcinoma') were collapsed into 'cancer.' Finally, groups 5 to 7 ('dementia,' 'chronic pulmonary disease,' and 'rheumatic disease') remained the same, while groups 8 ('peptic ulcer disease'), 12 ('paraplegia and hemiplegia'), 13 ('renal disease'), and 17 ('HIV/AIDS') were discarded, because there were fewer than five patients in each group.

### Statistical analysis

The association between suPAR and categorical variables was tested with a standard nonparametric one-way analysis (Kruskal-Wallis test), and the correlation between suPAR and the other biomarkers was tested with a Pearson correlation analysis. To study the adjusted effect of suPAR on mortality and readmission, we used Cox regression survival analysis with time to death and time to readmittance, respectively, as time variables, and death and readmittance, respectively, as censoring variables. When studying time to readmission, a competing risk model was used, and the patients were censored if they died within 30 days from discharge. The mean suPAR levels in the various modified Charlson groups were compared to the Charlson Score = 0 group with a *t*-test for equal or nonequal variances where appropriate. When studying the admission time, we used standard linear regression, where the 16 patients who died in hospital (and thus had a falsely short admission) were censored. The *P *values listed with the cumulative incidence plots are from a Cox regression survival analysis as above, but with the suPAR tertile or Charlson Score alone as the explanatory variable. We used the SAS 9.2 package (SAS Institute, Cary, NC, USA) for statistical analysis. The cumulative incidence plots were compiled with the SPSS 12.0 package (IBM, Armonk, NY, USA), and the scatterplot was compiled with GraphPad Prism 5.04 (GraphPad Software, San Diego, CA, USA).

## Results

### Baseline data

**Figure 1:**
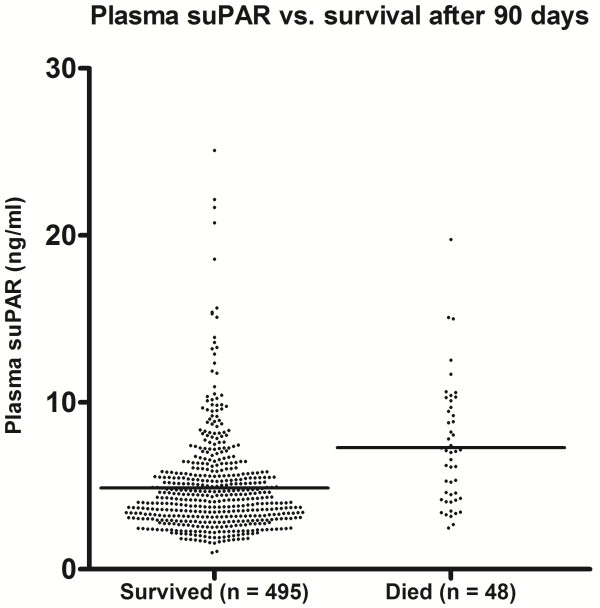
**Scatterplot showing the distribution of individual suPAR values among those who died within 90 days and those who survived for 90 days**. Horizontal lines are the mean suPAR values (4.87 ng/ml for survivors and 7.29 ng/ml for non-survivors).

During the two month inclusion period, 569 patients were enrolled. Of these, 20 had missing blood samples, two withdrew consent, and four patients emigrated during follow-up, leaving 543 patients for the final analysis. The patients' baseline characteristics are listed in Table 1. There were 46.4% men, and the median age was 68.8 years. The median suPAR level was 4.28 ng/ml (range 0.99 to 25.10 ng/ml), and there was no association between sex and plasma suPAR concentration (*P *= 0.53). See Figure 1 for distribution of individual suPAR values. As shown in Table 1, more than half of the patients scored 0 on the Charlson Score, while five patients scored 4 or more. In total, there were 219 different baseline ICD-10 diagnoses when not grouped.

**Table 1:** Baseline characteristics of the study cohort (n = 543).

Variable (unit)	*N*	Median or percent	2.5th Percentile	97.5th Percentile
Age (years)	543	68.8	24.2	92.7

Sex				

Male	252	46.4 %	-	-

Female	291	53.6 %	-	-

Baseline diagnosis				

Symptoms, signs or for observation^a^	126	23.2 %	-	-

Respiratory disease	112	20.6 %	-	-

Cardiovascular disease	88	16.2 %	-	-

Endocrine disease	43	7.9 %	-	-

Infectious disease^b^	36	6.6 %	-	-

Hematological disease	26	4.8 %	-	-

Other	112	20.6 %	-	-

Charlson Score				

0	342	63.0 %	-	-

1	152	28.0 %	-	-

2	35	6.45 %	-	-

3	9	1.66 %	-	-

4+	5	0.92 %	-	-

SuPAR (ng/ml)	543	4.28	1.81	13.6

Creatinine (μmol/l)	541	76.0	42.0	215

CRP (mg/l)	540	8.00	1.00	286

Leukocytes (10^9^/l)	539	8.30	3.60	22.9

ALAT (U/l)	541	22.0	8.00	120

Albumin (g/l)	538	35.0	22.0	43.0

Hemoglobin (mmol/l)	541	7.90	5.00	9.90

^a^ICD-10 Chapter XVIII diagnoses (R00-R99) and Chapter XXI diagnoses (Z00-Z99) collapsed; ^b^ICD-10 Chapter I diagnoses (A00-B99). Some values were missing for biomarkers other than suPAR (samples failed because of hemolysis or because they were lost).

In all, 1,072 patients were admitted to the Acute Medical Unit during the two-month study period, and thus 529 (49 %) were not included in the study. Analysis of anonymized data showed that these patients had a median age of 79.9 years versus 68.8 years for the study population. Of the patients not included in the study, 41.6 % were men while 46.4 % of the study population were men. A total of 59.0 % had a Charlson Score of 0, 28.9 % had a Charlson Score of 1, 8.51 % had a Charlson Score of 2, 2.08 % had a Charlson Score of 3, and 1.51 % had Charlson Score of 4+ (compare with Table 1).

### Correlation between suPAR and other biomarkers

Pearson correlation analysis between suPAR and the standard biomarkers taken at admission showed a significant and positive correlation between suPAR and CRP (r = 0.28, *P *< 0.0001), creatinine (r = 0.25, *P *< 0.0001), ALAT (r = 0.13, *P *= 0.0025) as well as leukocyte cell count (r = 0.16, *P *< 0.0001). In contrast, hemoglobin and albumin showed a significant negative correlation to suPAR (r = -0.26, *P *< 0.0001 and r = -0.50, *P *< 0.0001, respectively).

### Association of SuPAR with endpoints

SuPAR showed a positive association with age as well as Charlson Score (both *P *< 0.0001, Table 2). A high suPAR upon admission was associated with increased length of hospital stay (*P *< 0.0001, Table 2).

**Table 2:** Mean suPAR (ng/ml) values in the various groups.

Variable	*N*	Mean	SD	*P*
Age				

18 to 50 years	114	3.66	2.28	

50 to 70 years	167	5.05	3.61	

More than 70 years	262	5.72	2.85	<0.0001

Sex				

Male	252	5.14	3.14	

Female	291	5.04	3.07	0.53

Admission time				

0 days	98	3.72	1.82	

1 day	189	4.50	2.57	

2 to 7 days	159	5.53	2.59	

8 days or more	81	7.06	4.83	<0.0001

Charlson Score				

0	342	4.70	2.84	

1	152	5.15	3.11	

2	35	7.24	3.85	

3	9	8.00	3.51	

4	5	8.77	2.67	<0.0001

Readmitted				

No	432	5.03	3.14	

Yes	111	5.31	2.96	0.24

Died within 30 days				

No	516	4.97	3.01	

Yes	27	7.31	3.89	0.0002

Died within 90 days				

No	495	4.87	2.96	

Yes	48	7.29	3.65	<0.0001

*P *values calculated with the Kruskal-Wallis test. SD indicates standard deviation.

**Figure 2:**
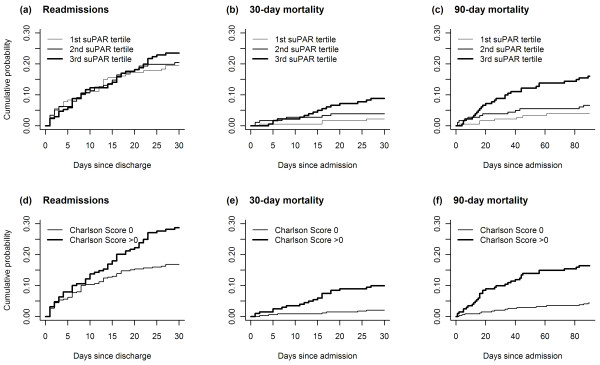
**Cumulative incidence plots of readmissions and 30- and 90-day mortality**. (a) Readmissions (*P *= 0.67), (b) 30-day mortality (*P *= 0.018), and (c) 90-day mortality (*P *= 0.0003) according to suPAR tertiles (n = 181 in each tertile). (d) Readmissions (*P *= 0.02), (e) 30-day mortality (*P *= 0.002), and (f) 90-day mortality (*P *< 0.0001) according to a Charlson Score of zero (n = 342) or more than zero (n = 201).

With regard to readmission, no association between baseline suPAR and readmission within 30 days was observed (Table 2 and Figure 2a). A higher Charlson Score was moderately associated with readmission (Figure 2d), and when adjusted for sex and age, the Hazard Ratio (HR) was 1.37 per 1 point increase in the Charlson Score, *P *= 0.03.

High baseline suPAR and a Charlson score of 1 or more was associated with increased 30-day (Figure 2b and 2e) and 90-day (Figure 2c and 2f) mortality. The mortality risk in the highest suPAR tertile was roughly similar to the mortality risk when having a Charlson Score of 1 or more. Baseline suPAR among 30-day (from admission) survivors was 4.97 ng/ml compared to 7.31 ng/ml among those who died (n = 27). Similarly, suPAR was higher in those who died within 90 days (n = 48, 7.29 ng/ml) compared to those who survived over the same period of time (4.87 ng/ml). Furthermore, to determine whether suPAR was associated with mortality among patients who were quickly discharged, we carried out a subgroup analysis of patients who were discharged within 48 hours of arrival at the hospital (n = 289, 53%). Of these 289 patients, 12 were registered as dead 90 days after admission. Patients who died during the 90 days of follow-up had significantly higher suPAR (6.16 ng/ml) compared to 90-day survivors (4.15 ng/ml, *P *= 0.003). Similarly, the 16 patients who died during admission had a higher suPAR (6.92 ng/ml) compared to the 527 who either survived their admission or the entire follow-up period (5.03 ng/ml, *P *= 0.0008).

### Cox regression analyses

To model the association between suPAR and outcomes while adjusting for confounders, we carried out Cox regression analyses. When adjusting for age and sex, the HR mortality estimate for each 1 ng/ml increase in suPAR was 1.14 and 1.15 for 30- and 90-day mortality, respectively (both *P *< 0.001). The HR of suPAR remained significant after further adjustment for C-reactive protein (CRP) (1.11 and 1.13 for 30- and 90-day mortality, respectively) and the Charlson Score (1.10 and 1.11 for 30- and 90-day mortality, respectively) (Table 3).

**Table 3:** Hazard ratio for the listed outcomes per 1 ng/ml increase in suPAR when adjusting for age, sex, CRP, and Charlson Score.

Adjustments	Readmitted within 30 days	Died within 30 days	Died within 90 days
Age and sex	1.01 (0.95 to 1.07)	1.14 (1.06 to 1.23)	1.15 (1.08 to 1.22)

Age, sex, and CRP	1.02 (0.96 to 1.08)	1.11 (1.02 to 1.20)	1.13 (1.06 to .20)

Age, sex, and Charlson Score	0.99 (0.93 to 1.06)	1.10 (1.01 to 1.20)	1.11 (1.04 to 1.19)

95% confidence intervals in parentheses.

### SuPAR in various Charlson groups

To further characterize the interaction between suPAR and the Charlson Score, we calculated mean suPAR values for all modified Charlson groups and compared them to those with a Charlson Score of zero (Table 4). Cancer, liver disease, diabetes, and cardiovascular disease appeared to increase suPAR, while dementia and chronic pulmonary disease (CPD) did not. Patients in the rheumatic disease group tended to have a higher suPAR, but the difference was not significant.

**Table 4:** Mean suPAR values in the various modified Charlson groups and for those not in a group (Charlson Score = 0).

Charlson group	Charlson Score 0	Cardiovascular disease	Dementia	Chronic pulmonary disease	Rheumatic disease	Diabetes	Liver disease	Cancer
*N*	342	50	10	81	6	54	8	17

SuPAR (SD)	4.70 (2.84)	5.88 (3.39)	4.53 (2.38)	4.96 (2.77)	5.98 (1.16)	6.06 (2.91)	11.8 (8.31)	8.12 (2.79)

*P*		0.0081	0.85	0.47	0.28	0.0013	0.046	<0.0001

*P *value is for a *t*-test of the group compared to those not in a group. SD, standard deviation.

To investigate the association between suPAR and admission time, we carried out a linear regression of admission time on age, sex, Charlson Score, and suPAR. The SuPAR beta-value was 0.45 (*P *< 0.0001). In other words, a 1 ng/ml increase in suPAR on average increased the admission length by 0.45 days when adjusting for age, sex, and Charlson Score.

## Discussion

### SuPAR as a potential disease severity marker

The association between increased suPAR and mortality has been extensively documented [7]. However, most of the studies were confined to healthy subjects [12] or subjects with well-defined illnesses, such as tuberculosis [3] or HIV [15]. In this study, we aimed to confirm suPAR's association with mortality in a more heterogeneous cohort, but also to investigate if suPAR is a disease severity marker in a broader sense through association with comorbidity burden (Charlson Score), admission length and readmission rates. We found that suPAR showed a dose-response relationship with the Charlson Score, was strongly associated with 30- and 90-day mortality, even when adjusting for age, sex, Charlson Score and CRP, but not associated with readmission rates. Given that the Charlson Score is moderately associated with readmission (HR 1.37) and strongly associated with suPAR, the lack of association between suPAR and readmissions is somewhat surprising. Taken together, the association with higher mortality, longer admissions, and higher comorbidity burden suggest that suPAR actually reflects general disease severity.

### SuPAR's correlation with other biomarkers and the modified Charlson groups

We found a significant positive correlation between suPAR and CRP, creatinine, ALAT, and leukocytes while there was a significant negative correlation between suPAR, hemoglobin, and albumin. These associations seem to support earlier studies. Elucidating on the kinetic relation of the pathophysiology within these associations is beyond the design of this study.

Both CRP and suPAR rise as part of an acute phase response, but CRP is produced by hepatocytes [16] whereas suPAR is produced peripherally mainly by leukocytes and activated endothelium [1], which may explain the positive association with leukocyte counts. Regarding the inverse relationship with albumin and hemoglobin, we interpret this as responses to chronic disease, for example, advanced cancer: it causes cachexia and malnutrition (low hemoglobin and albumin) as well as a systemic inflammation (high suPAR). There may be more direct signaling pathways involved, but our design makes it impossible to elucidate this further.

Regarding the nature of the association with creatinine, this is in agreement with Koch *et al*. who reported a similar correlation [6]. Also, there are reports that suPAR may even cause the kidney disease, focal segmental glomerulosclerosis (FSGS). Wei and coworkes recently showed that suPAR can enter the kidney glomerulus and bind and activate the β3 integrin, one of the major proteins anchoring podocytes to the glomerular basement membrane, and that increased plasma levels of suPAR lead to increased β3 integrin activation resulting in podocyte dysfunction and proteinuria [17]. However, none of the patients in this study were suffering from glomerular disease and it is currently unknown if suPAR has an active role in disease outside FSGS. Two earlier studies found a strong connection between high suPAR and decreased liver biosynthesis and cirrhosis [6,18], and the association between ALAT and suPAR may support these findings: also, patients in the Charlson group 'Liver disease' had a mean suPAR of 11.8 ng/ml, the highest mean value among the groups. If suPAR does not play an active role in disease outside FSGS, it is likely that the suPAR level reflects a conglomerate of negative biological processes such as increased inflammation and fibrosis, organ dysfunction and decreased organ biosynthesis.

### SuPAR--a true prognostic marker?

The Charlson Score is known to perform well when predicting mortality [10], but requires diagnostic information not always available upon admission, although this may be the case for more chronic patients. Based on adjusted analysis, suPAR levels appear to add information about disease severity that cannot be explained by the patient's sex, age, and diagnoses alone. However, as it also clear that interpretation of lone suPAR values is very difficult (see Figure 1), we propose that suPAR may be valuable as an addition to a prognostic model. Because of this population's heterogeneity and small size, we cannot make sound suggestions for actual cut-off values. Based on our results, such a prognostic model should include age, sex, suPAR, and, ideally, Charlson Score. A large prospective intervention trial is needed to evaluate the performance of such a model in actual clinical decision making, for example, whether to admit a patient or not.

### Limitations of the study

We included half the total number of patients admitted to the Acute Medical Unit during the two month study period. Gaps in the inclusion on Fridays and Saturdays account for a fair proportion of these as patients. We did not include patients on Fridays and Saturdays as the protocol stated that plasma had to be separated from blood within 24 hours, a job that was carried out by a laboratory technician working on weekdays. Also, a large proportion of the patients met exclusion criteria or was unable to understand or sign the consent form in an ethical manner. Less than one patient per day refused to participate. According to an analysis of the unincluded patients, the study population was 11.1 years younger, but had a similar distribution of Charlson Scores and sex as the unincluded patients. There was a potential bias towards exclusion of the oldest and most ill, as these patients were not always able to sign or understand the consent form. If this were to change our results, we expect that the associations found would be similar, but with greater power as there would probably be more fatality cases.

The validity of using ICD-10 codes from the Danish Patient Registry for calculating the Charlson Score has been documented in a large Danish cohort by Thygesen *et al*. who found an overall positive predictive value (PPV) of 98% for the Charlson groups [19]. However, although the diagnoses from the National Patient Registry are validated for calculating the Charlson Score with a high PPV, they are more inaccurate individually. This is because the Charlson Score only incorporates more serious conditions which are more likely to be coded correctly, and affiliated diagnoses are grouped together in the Charlson groups (for example, diabetes with complications), rendering discrimination between these superfluous. Moreover, subgroup analysis based on ICD-10 diagnoses would require that we choose a main diagnosis among others based on clinical experience; the Charlson Score does this automatically, and thus the results are more reproducible. Another limitation of the study was that we did not have sufficient data to calculate SAPS, Sequential Organ Failure Assessment (SOFA) or APACHE scores. Previous studies have shown that Charlson Score has similar prognostic value, and the association between comorbidites and suPAR was the main purpose of this work. In an earlier study, suPAR and age performed similarly to these physiologic scoring systems in a cohort of 151 systemic inflammatory response syndrome (SIRS) patients [20].

## Conclusions

We found that plasma suPAR concentrations in a heterogeneous cohort of patients with various diseases are not associated with readmission rates, but they are strongly associated with admission time, as well as mortality and the Charlson Comorbidity Index Score. Hence suPAR is not only associated with increased mortality, but it is a marker of overall disease severity.

## Key messages

• suPAR is associated not only with mortality, but also with admission time and the Charlson Comorbidity Index Score.

• suPAR is therefore a marker of disease severity in general.

• The findings apply to a diverse patient population.

## Abbreviations

APACHE: Acute Physiology and Chronic Health Evaluation; Charlson Score: Charlson Comorbidity Index Score; COPD: chronic obstructive pulmonary disease; CPD: chronic pulmonary disease; CRP: C-reactive protein; ELISA: enzyme-linked immunosorbent assay; FSGS: focal segmental glomerulosclerosis; HR: hazard ratio; ICD-10: International Classification of Diseases: 10^th ^Edition; PPV: positive predictive value; SAPS: Simplified Acute Physiology Score; SD,standard deviation; suPAR: soluble urokinase plasminogen activator receptor; uPAR: urokinase plasminogen activator receptor.

## Competing interests

JE-O is a co-founder and shareholder in ViroGates A/S, the company that produces the suPARnostic^® ^assay. JE-O and OA are inventors on a patent on suPAR and prognosis owned by Copenhagen University Hospital Hvidovre. The other authors declare that they have no competing interests.

## Authors' contributions

TH is the main author of the manuscript and designed the study. JP is responsible for all statistical analysis as well as close revisions of the manuscript. OA and J-EO took intiative in the study as well as contributed to the manuscript. CT measured samples and contributed with revisions of the manuscript. HK contributed with revisions of the manuscript. GE is the head of the Acute Medical Unit; she provided access to the patients as well as revisions of the manuscript. All authors have read and approved the final manuscript.
